# The Effect of Prosthetic Alignment on Prosthetic and Total Leg Stiffness While Running With Simulated Running-Specific Prostheses

**DOI:** 10.3389/fspor.2019.00016

**Published:** 2019-08-22

**Authors:** Ashley Groothuis, Han Houdijk

**Affiliations:** ^1^Department of Human Movement Sciences, Faculty of Behavioral and Movement Sciences, Vrije Universiteit Amsterdam, Amsterdam Movement Sciences, Amsterdam, Netherlands; ^2^Department of Research and Development, Heliomare Rehabilitation, Wijk aan Zee, Netherlands

**Keywords:** lower limb amputation, prosthesis, stiffness, running, spring-mass model

## Abstract

Running-specific prostheses (RSP) are designed to replicate the spring-like behavior of the biological leg in people with a lower limb amputation. Running performance strongly depends on stiffness of the RSP. The aim of this study was to investigate the effects of angle of alignment of the RSP on its stiffness, and how this affects total leg stiffness and the gait pattern during running. Ten able-bodied athletes performed eight trials on a treadmill with running-specific prosthetic simulators, while the alignment of the blades relative to the socket was set in four different angles (0, 5, 10, and 15°) during two different step frequency conditions (free and imposed). RSP stiffness, total leg stiffness, residual leg stiffness, and spatiotemporal parameters were measured. In both step frequency conditions, the RSP stiffness decreased linearly with increasing angle of alignment. Able bodied athletes were able to compensate for the decreased RSP stiffness, and keep total leg stiffness almost invariant, by increasing residual leg stiffness through a more straight the knee at initial contact. This study confirms that alignment is an important factor to take into account when optimizing the RSP. Whether the observed compensations are feasible in amputee athletes needs further investigation.

## Introduction

**Figure 1:**
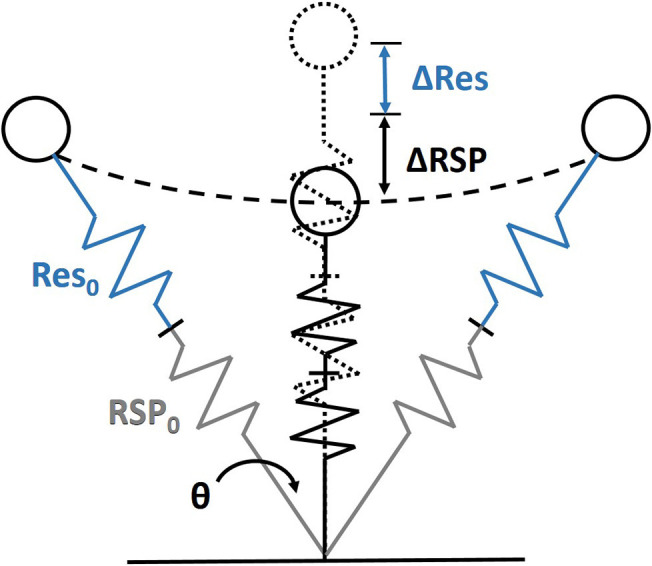
The spring-mass model for runners with lower leg amputation consisting of a point mass, representing body mass, and two linear springs connected in series, representing the behavior of the residual leg (Res) and running-specific prosthesis (RSP). Angle of attack is indicated by θ. During midstance the initial leg length (Res_0_ + RSP_0_) compresses (ΔRes + ΔRSP).

Athletes with a lower limb amputation use prostheses that mimic the spring-like behavior of the biological human leg. Human running is seen as a bouncing gait for which the body can be modeled as a simplified spring-mass model (Blickhan, 1989; Farley et al., 1993). The spring-mass model consists of a mass equivalent to the body mass supported by a single linear spring (Figure 1). This model can give insight into the relation between properties or behavior of the leg and running performance (i.e. running velocity and efficiency). It has been shown that running performance depends strongly on step frequency (Morin et al., 2007; de Ruiter et al., 2014). Step frequency of a spring mass system depends on the stiffness of the leg and the angle of attack of the leg with the ground (Blickhan, 1989; Farley et al., 1998; Morin et al., 2007). Hence, to improve running performance, leg stiffness and landing angle are important parameters that could be manipulated.

Able-bodied runners have been shown to be able to adapt leg stiffness to change step frequency or to accommodate to different surface stiffness (Farley and Gonzalez, 1996; Morin et al., 2007; Hobara et al., 2013). Their total leg stiffness is regulated through individual joint stiffness and leg joint configuration (Farley and Gonzalez, 1996). For athletes with a running-specific prosthesis (RSP) the virtual leg spring consists of a two series-connected springs: one virtual spring representing the behavior of the residual leg, and one real spring representing the properties of the prosthesis (Figure 1). Hence, in case of the amputee runner, the total leg stiffness is depending on the stiffness and configuration of the residual joints together with the stiffness of the prosthesis (Nolan, 2008). Oudenhoven et al. (2017) investigated the individual contribution of RSP and residual leg stiffness to total leg stiffness, and assessed whether athletes using a RSP are also able to adjust the total leg stiffness by regulating the stiffness of the residual joint. Although stiffness of the RSP dominated total leg stiffness, the stiffness of the residual leg was shown to contribute significantly to total leg stiffness. Nevertheless, athletes using a RSP seemed not capable of regulating the stiffness of the residual leg to adjust the total leg stiffness (Oudenhoven et al., 2017). Hence, careful selection of the RSP stiffness is therefore crucial to optimize performance in amputee runners.

If the amputee athlete wants to improve the running performance and if we want to further understand the prosthetic function, it is very important to understand the stiffness properties of the prosthesis (Beck et al., 2016). Currently, athletes are assigned a stiffness category based on the body mass and intended activity level, rather than performance based metrics of the prosthesis (Baum et al., 2013; Beck et al., 2016). The exact stiffness of blade in each category is not always known. Moreover, it has been shown that the stiffness of the RSP is not a constant but depends on loading conditions. Dyer et al. (2014) showed, for instance, that prosthetic stiffness is force dependent. This means that the prosthetic stiffness decreases when the force with which it is loaded increases. Furthermore, it has been shown that by changing the direction of loading of the blade the prosthetic stiffness could be changed (Beck et al., 2016; Litzenberger et al., 2016). There are two ways that the direction of loading can be manipulated: (1) changing the angle of attack of the leg at initial contact, for example by changing step length or (2) changing the angle of alignment of the RSP beneath the socket. Beck et al. (2016) demonstrated that the angle of attack increases as a function of running speed. Concomitantly, it was shown, using an artificial test bench, that with such an increase in angle of attack the stiffness of the RSP decreased. In addition, Litzenberger et al. (2016) showed that the stiffness of the RSP decreased as the mounting angle of the blade relative to the socket increased. However, in both studies the prostheses were tested in isolation using a laboratory test bench with a rotating, low-friction base. This artificial set-up does not take into account the true dynamics of the running movement.

In the present study, we investigated the effect of alignment of the blade relative to the socket on prosthetic stiffness and total leg stiffness during actual running on a treadmill. We investigated this in two conditions; while running with either a fixed or free step frequency, both at a similar fixed running speed. The condition in which the step frequency is fixed was used to limit the possibility of changing step length and the angle of attack during running, which could confound the effect of alignment. In the condition in which the step frequency was free, step length and angle of attack could be chosen freely by the participant. In this condition, it could be investigated how participants adjust their gait pattern to changes in alignment, and therefore this condition could reveal how alignment changes will affect running in a real life situation. We hypothesized that prosthetic stiffness would decrease as a function of alignment angle, during both step frequency conditions (McGowan et al., 2012; Beck et al., 2016). Furthermore, we explored whether runners would adapt angle of attack during running (in the free step frequency condition) and/or whether runners would adapt residual leg stiffness to mitigate the potential change in prosthetic stiffness with changing alignment during running. The present study was performed with able-bodied participants using a set of prosthetic simulators, as a first step to explore these effects before applying these manipulations to actual athletes with a lower limb amputations. This study will reveal to what extend prosthetic alignment might affect prosthetic properties and hence performance in blade running.

## Methods

### Participants

Ten able-bodied endurance athletes were included (7 male/3 female, age 21 ± 1.8, height 1.77 ± 0.10 m, body mass 69.8 ± 9.0 kg). For this study, experimental running-specific prosthetic simulators (RSP) were developed. To make sure that the participant fitted the prostheses and were able to complete the experiment, the following inclusion and exclusion criteria were set: the participants age ranged between 18 and 50 years, weight between 60 and 85 kg, and shoe size was between 39 and 43. In addition, participants needed to be healthy without the use of medication for heart and lungs or physical injuries and they should be able to run for more than 10 min with the experimental prostheses. All participants provided written informed consent prior to the experiment. This study was approved by the local ethics committee.

### Experimental Prostheses

**Figure 2:**
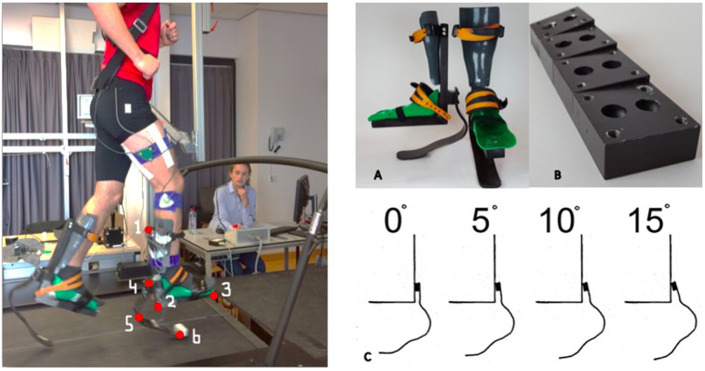
**Left**: Experimental setup. Virtual markers on the RSP: (1) proximal end of the L-frame, (2) 90 degrees angle of L-frame, (3) distal end of L-frame, (4) proximal end of the blade, (5) bend of the blade, (6) ground contact point during running. **Right**: (A) Prosthetic simulators. (B) Wedges to change angle of alignment of the blade. (C) Orientation of the blade relative to the orthosis in four different angles (0°, 5°, 10°, and 15°).

For this study a set of RSP simulators for able bodied athletes was developed (Figure 2). The prosthetic simulators consisted of an orthosis with a J-shaped Ottobock 1E90 blade attached to the back. The orthosis was made from two aluminum beams connected in a 90 degrees' angle, with two anatomically shaped shells, one for the foot and one for the shank, to provide wearing comfort. To rigidly fix the participants' foot and ankle two bindings were placed over the shin and feet. The alignment of the blade relative to the orthosis could be changed between 0 and 15°, in steps of 5 degrees (Figures 2B,C). The prosthetic simulators allowed a maximal running speed of 10 km/h and maximal body weight of 85 kg.

### Task and Procedures

The measurements were performed on a dual belt treadmill with embedded force plates (Ymill, Motekforce Link, The Netherlands). Participants first learned how to run on the RSPs, both overground and on the treadmill, during two practice sessions before the actual day of the experiment. In the final practice session, the preferred running speed (PS) on the treadmill was determined. On the day of the actual experiment, participants first started with a warming-up of 5 min to accommodate again to the prosthesis. During this warming-up, the preferred step frequency (PSF) was determined while the participants ran for 2 min at their preferred speed with a prosthetic alignment of 0°. During the experiment in total 8 trials were executed. Each participant was asked to run with 4 different angles of alignment of the blade relative to the orthosis (0, 5, 10, and 15°) in two different step frequency conditions: imposed and free step frequency. During the imposed condition the PSF at 0° was fixed for all al alignments using a metronome, while during the free condition participants were free to select a different step frequency for different alignments. The 8 experimental trials were divided in four blocks (each alignment) of two trials (respectively, the free and imposed condition trial). The order of the 4 blocks with different angles of alignment were randomized between participants. The duration of each trial was 2 min, with 5 min rest in between the trials and a short accommodation time with the new alignment before each trial. For safety, participants were supported by an overhead safety harness and were allowed to grab the horizontal bar in front of the treadmill in case they lost balance.

### Data Collection and Equipment

The embedded force plates in the treadmill were used to measure the ground reaction force (GRF, sample frequency 100 Hz). Motion analysis (Optotrak Northern Digital, Waterloo, Ontario, Canada, sample frequency 100 Hz) was used to measure the 3D-kinematics of blades and leg segments, with cluster markers on the pelvis, thigh, L frame of the orthoses and distal end of the RSP, only for the right leg. The following anatomical landmarks were captured in a static calibration trial: the tip of the toe, calcaneus, lateral and medial malleolus, lateral and medial epicondyle, trochanter major, left and right SIAS, midpoint between the left and right ASIS and the belly button. We additionally captured six landmarks on the RSP: three to indicate the corners of the L-frame of the orthosis and three to indicate the tip, bend and end of the blade (Figure 2).

### Data Analysis

#### Pre-processing

For all outcome measures 100 consecutive steps during the final minute of each trial, during which steady state running pattern was achieved, were analyzed and averaged. The raw kinematics and force plate data were filtered with a low pass 2nd order Butterworth filter, with a cut-off frequency of 20 Hz. Using a 3D inverse dynamics model the kinematics of the pelvis, thigh, shank, and foot were calculated and the net joint moments around knee were determined (Kingma et al., 1996). Anthropometric data of the inverse dynamics model was based on the data presented by Zatsiorski (1998) and Faber et al. (2011). Foot and blade were modeled as a lumped segment, with the mass of the prosthetic simulator added to the foot segment located in the center of mass (CoM) of the foot. This simplification had little impact on the knee joint moment as the moment of gravity on the foot-blade segment is negligible relative to the moment of the ground reaction force.

#### Outcome Measures

In this study, the total leg stiffness (K_tot_) and the stiffness of the RSP (K_RSP_) were the primary outcome measures. Total leg stiffness and RSP stiffness were determined along the line from center of pressure (CoP) to body CoM, which was approximated by the CoM of the pelvis (Oudenhoven et al., 2017). This one dimensional analysis fits best with the assumptions of the spring mass model that perceives the leg spring as a single linear spring running from point of attack to CoM. We decomposed the GRF vector, RSP length vector and total leg length vector in a component along the designated dimension and a component perpendicular to this dimension. Subsequently, we only analyzed the component along the designated dimension. This approach is more detailed but basically similar to most previous studies (e.g., Farley and Gonzalez, 1996; McGowan et al., 2012), who were generally forced to use only peak vertical ground reaction force and total leg length change at mid-stance to calculate leg stiffness, in the absence of recorded 3D ground reaction forces. Those studies assume that ground reaction force and leg angle are aligned and vertical in midstance. All stiffnesses were normalized to the body mass of the participant.

**Figure 3:**
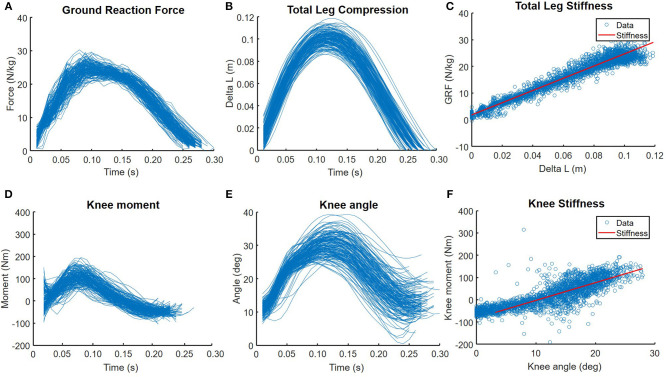
Example of 100 consecutive steps for **Upper**: **(A)** ground reaction force, **(B)** leg compression, **(C)** ground reaction force as a function of leg compression of one participant running with the RSP at imposed step frequency. Leg stiffness was calculated as a linear curve fit over the GRF as a function of leg compression, between the instant of touchdown and the instant at which maximal vertical GRF was reached. **Lower**: **(D)** knee moment, **(E)** knee angle, **(F)** knee joint moment as a function of knee angle. Knee stiffness was calculated as a linear curve fit over the knee moment as a function of knee angle, between the instant of touchdown and the instant at which maximal knee angle was reached.

K_tot_ was estimated with a linear curve fit over the data of GRF as function of the total leg compression (ΔL), between the instant of touchdown and the instant at which maximal vertical GRF was reached (Figures 3A–C). Total leg length was defined as the distance between the body CoM and the CoP of the GRF under the RSP. Total leg compression was calculated as the differences between total leg length at initial contact (L_0_) and total leg length throughout ground contact (Figure 1).

To estimate K_RSP_ we used a linear curve fit over the GRF as function of the compression of the RSP spring, between the instant of touchdown and the instant at which maximal vertical GRF was reached. The compression of the RSP spring was determined as the difference in length (RSP_0_) at initial contact and the length throughout ground contact. The distance between the most proximal point of the RSP spring and the CoP along the dimension of interest represented the length of the RSP spring.

The secondary outcome measures, to explore potential adaptation in the gait pattern of the participants in response to the alignment changes, were: step frequency (f_s_), stride time (t_s_), contact time (t_c_), flight time (t_f_), angle of attack of the leg (θ), and residual leg stiffness (K_res_). Average step frequency (steps/min) was calculated as the number of GRF peaks during the 2 min trials divided by 2. Contact time and flight time were determined with a vertical GRF threshold at 50 N. A vertical GRF above 50 N was set as contact time and a vertical GRF lower than 50 N was set as flight time. The stride time was set from initial contact till next initial contact of the right blade. K_res_ was calculated by: $\frac{1}{\text{Kres}}\;=\;\frac{1}{\text{Ktot}}-\frac{1}{\text{Krsp}}$. As mentioned above, K_tot_ and K_RSP_ was calculated with a linear curve fit. These outcome measures were used to calculate K_res_. In addition, knee joint stiffness (K_knee_) and knee angle at initial contact (φknee) were analyzed to explain potential changes in K_res_ (Farley et al., 1998). To calculate K_knee_ a linear curve fit was performed over the knee angular displacement as function of the net joint moment at the knee, between the instant of touchdown and the instant at which maximal knee angle was reached (Figures 3D–F). The knee flexion-extension angle was obtained from the Euler decomposition of the orientation matrix of shank relative to thigh. To obtain the knee angle at initial contact (φ_knee_) the first sample during every stance face was taken. Angular displacement of the knee was defined as the change in knee angle relative to the touchdown knee angle. The angle of attack (θ) was calculated as the angle between the total leg length vector at initial contact and the vertical (Figure 1).

#### Statistical Analysis

For the statistical analysis of the kinematic and kinetic parameters a one-way repeated measures ANOVA was used for each of the two step frequency conditions (free or imposed) separately. The within factor was angle of alignment (0, 5, 10, and 15°). In case there was a significant main effect of alignment, a *post hoc* analysis was performed to test for a significant linear contrast between angle of alignment and the dependent variable. Analyses were performed using IBM^©^ SPSS Statistics version 24 and significance was set at *p* < 0.05.

## Results

**Figure 4:**
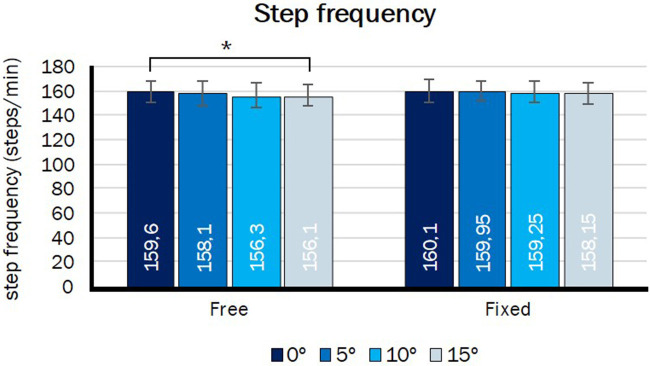
Step frequency in two step frequency conditions, Free and Fixed, over the four different alignment angles. ^*^Significant linear contrast of alignment angle (*p* < 0.05).

All 10 participants successfully performed all experimental conditions. During the imposed step frequency trials, all participants were able to synchronize average step frequency to the imposed PSF, within an error margin of 2.5%. Mean PSF was 161.1 steps/min (±10.3). The preferred speed ranged between 8.0 and 9.5 km/h. For the step frequency imposed condition, no significant difference in step frequency between the four angle of alignments was found (*p* = 0.225, Figure 4), while there was significant linear decrease from 160.1 ± 9.5 steps/min at 0° to 156.1 ± 8.8 steps/min at 15° in the step frequency free condition (*p* = 0.016, Figure 4). Due to technical issues two subjects needed to be excluded in the analysis of the knee angle at initial contact and one subject needed to be excluded in the analysis of the knee joint stiffness.

### Imposed Step Frequency Condition

#### RSP Stiffness and Total Leg Stiffness

**Figure 5:**
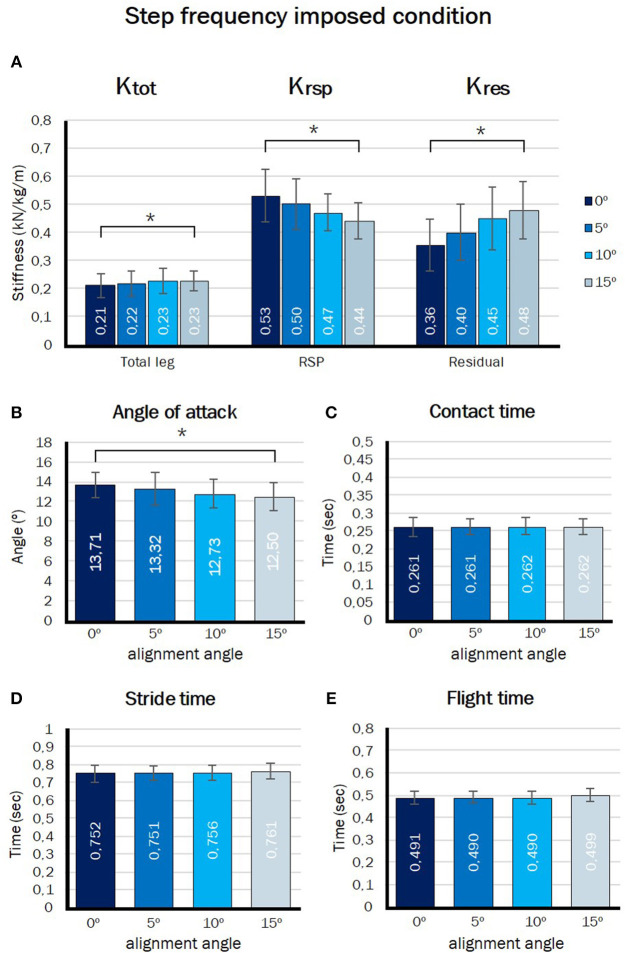
Outcome measures in fixed step frequency imposed condition. Effect of angle of alignment on **(A)** total leg stiffness, RSP stiffness, and residual leg stiffness, **(B)** angle of attack, **(C)** stride time, **(D)** contact time, and **(E)** flight time. ^*^Significant linear contrast of alignment angle (*p* < 0.05).

Figure 5A presents the effect of angle of alignment on the stiffness of the RSP (K_RSP_), stiffness of the residual leg (K_res_), and the total leg stiffness (K_tot_) at four different alignment angles during the step frequency imposed condition. The effect of angle of alignment was significant for all three stiffnesses calculated, K_tot_ (*p* = 0.011), K_RSP_ (*p* = 0.000), and K_res_ (*p* = 0.000). K_tot_ and K_res_ increased linearly (*p* = 0.025 and *p* = 0.000), whereas K_RSP_ showed a significant linearly decreasing contrast (*p* = 0.000) when the angle of alignment increased.

#### Angle of Attack and Step Frequency, Stride-, Contact-, and Flight Time

Figure 5B presents the effect of angle of alignment on angle of attack. There was a significant effect on angle of attack (*p* = 0.023). The angle of attack decreased linearly from 13.7 ± 1.3° to 12.5 ± 1.4° when the angle of alignment increased (*p* = 0.017). Figures 5C–E presents the effect of angle of alignment on stride-, contact- and flight time (t_s_, t_c_, and t_f_). During this condition the step frequency was imposed. No significant effect of angle of alignment was found for stride-, contact-, and flight time (respectively, *p* = 0.255, *p* = 0.891, and *p* = 0.152).

#### Knee Stiffness and Knee Angle at Initial Contact

The effect of angle of alignment on the knee joint stiffness (K_knee_) and knee angle at initial contact is shown in **Figure 7**. Changing alignment resulted in a significant linear decrease of the knee angle at initial contact (*p* = 0.003). The knee angle at initial contact decreased from 17.7 ± 5.2° to 13.3 ± 7.2°. There was no significant effect for the knee joint stiffness (*p* = 0.092).

### Free Step Frequency Condition

#### RSP Stiffness and Total Leg Stiffness During

**Figure 6:**
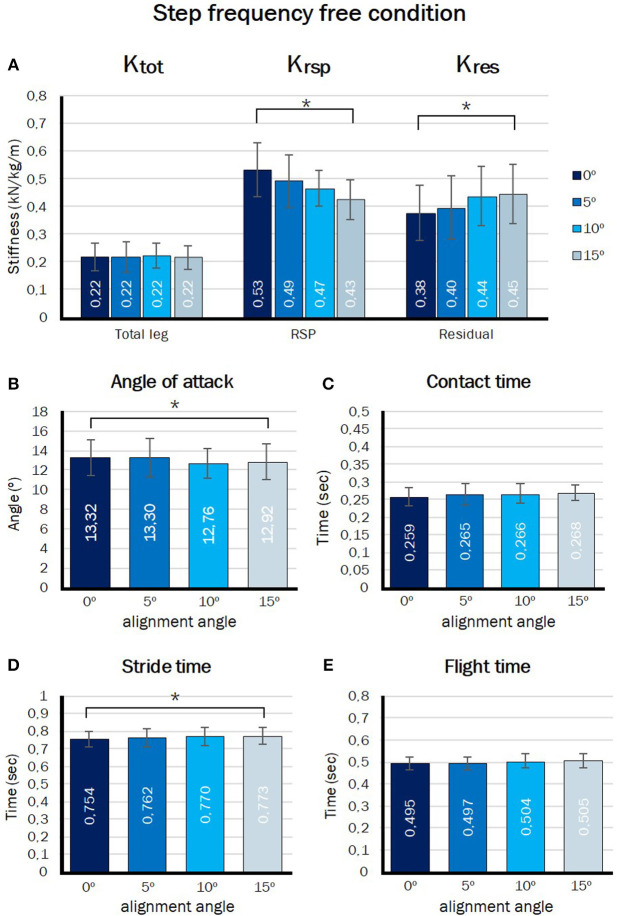
Outcome measures in step frequency free condition. Effect of angle of alignment on **(A)** total leg stiffness, RSP stiffness, and residual leg stiffness, **(B)** angle of attack, **(C)** stride time, **(D)** contact time, and **(E)** flight time. ^*^Significant linear contrast of alignment angle (*p* < 0.05).

The total leg stiffness, stiffness of the RSP and stiffness of the residual leg during the free step frequency condition are shown in Figure 6A. When the participant was free to choose the step frequency the manipulation of angle of alignment resulted in a significant different RSP stiffness (*p* = 0.000) and residual leg stiffness (*p* = 0.002), but there was no significant effect for the total leg stiffness (*p* = 0.946). The RSP stiffness showed a significant linear decrease while the alignment angle increased (*p* = 0.000), whereas the residual leg stiffness showed a significant linear increase (*p* = 0.001).

#### Angle of Attack and Step Frequency, Stride-, Contact-, and Flight Time

Figure 6B shows the effect of angle of alignment on the angle of attack in free step frequency condition. The manipulation of angle of alignment results in a significant effect on the angle of attack (*p* = 0.027). The angle of attack significantly decreased from 13.3 ± 1.8° to 12.9 ± 1.8° when the angle of alignment increases (*p* = 0.006). Figures 6C–E show the effect of angle of alignment on stride-, contact-, and flight time (t_s_, t_c_, and t_f_). Stride time increased linear (*p* = 0.013) from 0.75 ± 0.05 s to 0.77 ± 0.05 s when the angle of alignment increased. Contact time tended to increase with alignment angle but this effect did not reach significance (*p* = 0.062), although *post hoc* a significant linear contrast could be found. Flight time did not change significantly (*p* = 0.205).

#### Knee Stiffness and Knee Angle at Initial Contact

**Figure 7:**
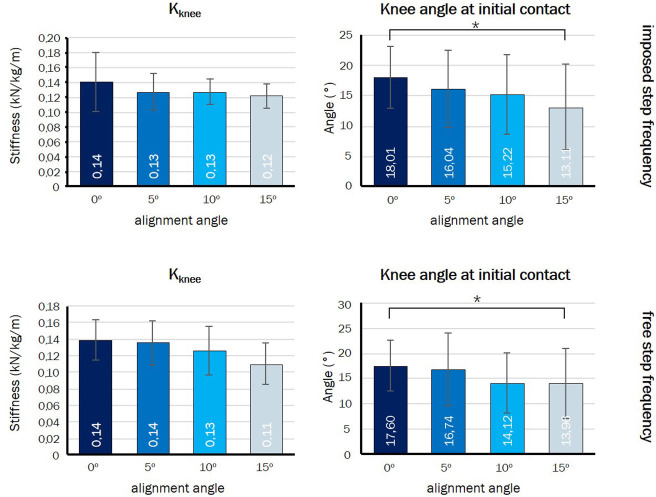
Knee stiffness and knee angle at initial contact in the imposed step frequency condition (**Upper**) and in the free step frequency condition (**Lower**). ^*^Significant linear contrast (*p* < 0.05).

Figure 7 presents the effect of alignment angle on the knee angle at initial contact and the knee joint stiffness during the step frequency free condition. Increasing the angle of alignment had a significant effect on the knee angle at initial contact (*p* = 0.003). The knee angle showed a significant decrease (more knee extension) from 18.0 ± 5.0° to 13.1 ± 7.1°. For the knee joint stiffness, no significant difference between the four angles of alignments was found (*p* = 0.355).

## Discussion

The primary aim of this study was to investigate whether the RSP stiffness depends on the angle of alignment of the RSP and how this affects total leg stiffness and running pattern. This effect was tested in two conditions, (1) a step frequency imposed condition to assess the effect of alignment independently from possible changes in leg angle of attack and (2) a step frequency free condition to include potential adaptations of the athletes to this manipulation in terms of changing leg angle of attack and gait pattern. The results reveal that the effects of angle of alignment in both conditions was almost similar. Therefore, we'll first discuss the general effects of angle of alignment and subsequently discuss the specific differences in the effects in both step frequency conditions.

### Effect of Alignment Angle on K_RSP_

As hypothesized, the stiffness of the RSP showed a significant linear decrease when the angle of alignment increased during both step frequency imposed and free condition (Figures 5A, 6A). Stiffness of the RSP decreased by 17% over the 15° alignment change in the step frequency imposed condition and by 19% in the step frequency free condition. Beck et al. (2016) and Litzenberger et al. (2016) also found that the RSP stiffness was lower at a larger loading angle in a test bench, which agrees with our results. Litzenberger et al. (2016) found an decrease of 31.81% over 18° increase in alignment angle. This difference in magnitude of the effect relative to our study might be attributed to different loading angles of the blades between studies and/or differences in blade types used. According to Beck et al. (2016) every 1° increase in loading angle of the type of blade used in our study caused a 0.45 kN/m decrease in the RSP stiffness when tested in a test bench. This magnitude is in line with the effect of alignment we observed during actual running.

An important limitation of previous studies (Beck et al., 2016; Litzenberger et al., 2016) was the fact that loading of the blades occurred in isolated artificial test bench conditions. These conditions do not sufficiently take into account the dynamics of actual running. Loading rates in the test bench were much lower compared actual running and potential changes in loading angle throughout the stance phase were not taken into account. Our study confirms, however, that the observed effects in a test bench set up can be reproduced during actual running. Hence, we can conclude that the actual stiffness of an RSP is substantially depending on the angle of alignment of the RSP during running. This effect of alignment should be taken into account when selecting the right blade for each athlete.

### Effect of Alignment Angle on K_tot_ and K_res_

For the second aim of this study, we explored how the runner would adapt total leg stiffness and the gait pattern to the potential change in RSP stiffness following the alignment change. Despite the considerable decrease in RSP stiffness there was no significant effect of alignment angle on the total leg stiffness in the step frequency free condition. In the fixed step frequency condition a constant total leg stiffness might be expected, as step frequency is closely related to leg stiffness. Surprisingly, in that condition we observed even a small, but significant, linear increase in leg stiffness as the alignment angle increased. Apparently, stiffness of the residual leg was voluntarily increased in response to a change in RSP stiffness in both conditions such that total leg stiffness did not decrease along with that of the RSP. This strategy has previously also been demonstrated in able-bodied athletes who were shown to adapt leg stiffness to changes in surface stiffness in manner that total stiffness (of leg and surface) remains constant (Farley et al., 1998; Ferris et al., 1998). In our study, the RSP could be seen as the surface people are running on. It appears that, also in this condition, participants compensated for the lower RSP stiffness as a function of alignment angle by increasing stiffness of the residual leg in order to keep total leg stiffness more or less constant.

Leg stiffness depends on joint stiffness and leg geometry. Able-bodied athletes, running on compliant surfaces, were shown to increase leg stiffness by increasing ankle joint stiffness and by landing with a more extended knee joint at initial contact (Farley et al., 1998). In our experimental setup up (nor in real amputee athletes) ankle joint stiffness could not be adapted as it was rigidly fixed in the prosthetic simulators. Instead, we observed that our participants increased residual leg stiffness through a significantly more extended knee joint at initial contact. The knee joint stiffness showed no significant change. This strategy could account for the change in residual leg stiffness and constant or even slight increase in total leg stiffness that we observed in this study.

The strategy and ability to increase K_res_ in order to keep K_tot_ constant used by the able-bodied participants using the RSPs in this study, is in contrast to the findings in a study of Oudenhoven et al. (2017). They observed that lower limb amputees did not seem able, or did not choose, to change residual leg stiffness when asked to change step frequency while running. It can therefore be argued whether athletes with a lower limb amputation have the same capacity to regulate residual leg stiffness compared to the able-bodied athletes in this study. Possibly strength in the residual leg muscles or issues related to prosthetic socket fit might limit their capacity to regulate residual leg stiffness. Consequently, generalization of the response found in this study to amputee athletes should be taken with care.

### Effect of Step Frequency

In this study we included a condition to keep step frequency (and hence step length) constant as we expected that the effect alignment might be confounded by gait adaptations, such as changes in loading rate, step length, and angle of attack. Surprisingly, very little changes in the gait pattern between the step frequency free and imposed conditions were observed. This can be explained by the fact that participants compensated for the considerably decrease of RSP stiffness by increasing residual leg stiffness in both the free and imposed step frequency condition. Small differences between these conditions might be attributed to the influence of the alignment angle on the angle of attack.

When the position of the tip of the blade relative to the socket rotates backwards, when the alignment angle is increased, the total leg vector (from CoP to CoM) becomes oriented more vertical when no additional changes in joint configurations are made. Indeed the angle of attack decreased slightly with increasing alignment angle in our experiments. According to the spring-mass model step frequency is a function of total leg stiffness and angle of attack (Blickhan, 1989; Farley et al., 1998; Morin et al., 2007). Following Farley and Gonzalez (1996) and Ferris et al. (1998), a decrease in the angle of attack results in a lower step frequency and longer contact time, while an increase in leg stiffness results in a higher step frequency and shorter contact time. Since the participants were asked to keep the step frequency constant in the imposed step frequency condition, they needed to increase their total leg stiffness to compensate for the decrease in angle of attack. This was indeed observed in the imposed frequency trials. During the step frequency free condition the angle of attack also decreased, but in this condition the participants did not have to keep the step frequency constant. As a consequence, the contact time increased and the step frequency decreased despite a constant total leg stiffness in this condition (Figures 4, 6). This could thus be attributed to the slight decrease in angle of attack.

### Limitations

There are some limitations in this study that should be taken into account when interpreting our findings. First, we included a relatively small number of participants in our study who were not used to running with prosthetic simulators. Participants only had two practice sessions to get used to running on the RSP with different alignment angles. Although all participants felt comfortable with running on the prosthetic simulators, it might take more time to really master this skill. This may affect the current results by introducing greater variability in the running pattern within and between participants. Nevertheless, our set up seems sufficient to reveal the mechanical effects of alignment on RSP stiffness during running. The effects of alignment on RSP stiffness were clear and consistent between trials and could be reproduced in both step frequency conditions. Moreover, the results confirm previous studies that investigated this topic in controlled bench test environments.

Finally, and most importantly, we did not include athletes with a lower limb amputation but able-bodied participants using prosthetic simulators. While we believe that the direct mechanical effects of alignment on RSP stiffness, as shown in this study, are independent of study population, the observed gait adaptations might not be similar. As shown previously by Oudenhoven et al. (2017), amputee athletes might not be able to increase residual leg stiffness and keep total leg stiffness constant between alignments. Future studies are therefore required to investigate the response of amputee athletes to the changes of RSP stiffness that accompany alignment changes.

## Conclusion

Prosthetic alignment substantially affects prosthetic stiffness and imposes gait adaptation to the user. Thus, if an optimal prosthetic stiffness is selected for the amputee athlete not only finding the right RSP stiffness category is essential but also the alignment of the blade relative to the socket needs to be taken into account. The specific gait adaption of lower limb amputees to changes in RSP stiffness that accompany alignment changes need further investigation.

## Data Availability

Data from this study are available at: https://dataverse.nl/dataset.xhtml?persistentId=hdl:10411/0JFYGF.

## Ethics Statement

All participants provided written informed consent prior to the experiment. This study was approved by the local ethics committee of the Faculty of Behavioral and Movement Sciences of Vrije Universiteit Amsterdam.

## Author Contributions

AG participated in the design of the study, carried out the data collection, participated in data analysis, and drafted the manuscript. HH participated in design of the study, participated in data analysis, and critically revised the manuscript. All authors gave final approval for publication and agree to be held accountable for the work performed therein.

### Conflict of Interest Statement

The authors declare that the research was conducted in the absence of any commercial or financial relationships that could be construed as a potential conflict of interest.
